# Management of Cytomegalovirus, Epstein-Barr Virus, and HIV Viral Load Quality Control Data Using Unity Real Time

**DOI:** 10.1128/JCM.01675-21

**Published:** 2022-01-19

**Authors:** Duane W. Newton, Nico Vandepoele, John C. Yundt-Pacheco, David Gauthier, Michael A. Bachman

**Affiliations:** a NaviDx Consulting, Mount Prospect, Illinois, USA; b Bio-Rad Laboratories, Irvine, California, USA; c Department of Pathology, Michigan Medicine, Ann Arbor, Michigan, USA; St. Jude Children's Research Hospital

**Keywords:** viral load, quality control, quality data management, total allowable error

## Abstract

Quality control (QC) rules (Westgard rules) are applied to viral load testing to identify runs that should be reviewed or repeated, but this requires balancing the patient safety benefits of error detection with the cost and inefficiency of false rejection. In this study, we identified the total allowable errors (TEa) from the literature and utilized a commercially available software program (Unity Real Time; Bio-Rad Laboratories) to manage QC data, assess assay performance, and provide QC decision support for both FDA-approved/cleared (Abbott cytomegalovirus [CMV] and HIV viral load) as well as laboratory-developed (Epstein-Barr virus [EBV] viral load) assays. Unity Real Time was used to calculate means, standard deviations (SDs), and coefficient of variation (CV; in percent) of negative, low-positive, and high-positive control data from 73 to 83 days of testing. Sigma values were calculated to measure the test performance relative to a TEa of 0.5 log_10_. The sigma value of 5.06 for EBV predicts ∼230 erroneous results per million individual patient tests (0.02% frequency), whereas sigma values of >6 for CMV (11.32) and HIV (7.66) indicate <4 erroneous results per million individual patient tests. The Unity Real Time QC Design module utilized these sigma values to recommend QC rules and provided objective evidence for loosening the laboratory’s existing QC rules for run acceptability, potentially reducing false rejection rates by 10-fold for the assay with the most variation (EBV viral load). This study provides a framework for laboratories, with Unity Real Time as a tool, to evaluate assay performance relative to clinical decision points and establish optimal rules for routine monitoring of molecular viral load assay performance.

## INTRODUCTION

Ensuring quality in molecular diagnostic assays employs a multipronged approach that can include validation and verification of new tests, regular monitoring of assay quality control reagents, routine utilization of independent controls to assess ongoing assay performance, participation in proficiency testing programs, and periodic competency assessments. While these activities assist in establishing and maintaining analytical performance, several challenges exist when attempting to ensure high-quality clinical performance for many molecular assays used for infectious disease diagnosis and monitoring. Although numerous WHO international standards exist for targets of molecular viral load assays (https://www.nibsc.org/), relatively few commercial assays utilize control reagents that are traceable to these standards, and lack of assay commutability exists, such that two assays with controls traceable to an international standard may give different results (1, 2). Furthermore, commutability can be influenced by differences in design and performance of in-kit controls from different commercial assay manufacturers despite their traceability to international standards. Finally, lack of commutability for viral load assays such as for BK virus, cytomegalovirus (CMV), and Epstein-Barr virus (EBV) hampers the development of objective clinical cutoffs for making management decisions and limits the ability of laboratorians to establish total allowable error (TEa), effectively the “tolerance limits,” for these assays. Consequently, laboratories are continually forced to reestablish “standards” that are applicable only to their laboratory and patient population.

Sigma metrics—quality management techniques traditionally used to identify and reduce defects in processes—are commonly used in clinical chemistry laboratories to monitor quantitative assays. These statistical tools have also recently been applied to viral load testing to attempt to define the QC parameters for assessing test performance as described by Westgard and Lucic (3). These authors described their approach for identifying defects, which for these purposes are viral load results that deviate from established tolerance limits. The number of defects observed over time can be reported on a scale of defects per million opportunities (DPMO), with processes functioning at “world class” levels of performance generating 3.4 or fewer DPMO (3–5). In diagnostics laboratories, it is not practical to count defects, so the sigma metric is calculated as (TEa [%] − bias [%])/CV [%], where TEa is the total allowable error (a clinically relevant change) and the CV (coefficient of variation) and bias are determined by repeatedly testing QC material or by comparing internal data to peer group or proficiency testing surveys (6).

Robust analytical performance of viral load assays is required to ensure the ability to detect clinically relevant changes in values—i.e., to ascertain that a significant change in a patient’s viral load over time is due to clinical factors (e.g., disease progression, antiviral resistance, or response to therapy) and not analytical factors caused by excessive assay variability (due to issues such as pipetting errors, loss of calibration, or instrument malfunction). One part of assay quality assurance—routine monitoring of in-kit and independent assay controls for acceptable performance—works to ensure that any excessive assay variability is identified and prevented. Although this process is required by regulatory agencies, the criteria for determining acceptability can vary and are often left to the laboratory to establish. Although sigma metrics provide an objective framework for assessing assay performance, there are relatively few scenarios where clinically relevant changes in viral load have been established in practice guidelines (e.g., HIV, hepatitis B virus [HBV], and hepatitis C virus [HCV]) (7–11), https://www.hcvguidelines.org/). In addition, capturing and managing molecular QC data have historically been manual and labor-intensive processes, relying on paper documentation or in-house-developed spreadsheets/databases. In the present study, we assessed how Unity Real Time (Bio-Rad Laboratories, Hercules, CA), a commercially available, modular QC data management software program, can be utilized to manage QC data, assess assay performance, and provide QC decision support through its QC Design module for both well-defined FDA-approved/cleared assays and laboratory-developed tests.

## MATERIALS AND METHODS

### Data collection and clinical setting.

The Clinical Microbiology Laboratory at Michigan Medicine is a high-complexity, academic laboratory performing viral load testing on the Abbott m2000 system. Assays for HIV and CMV viral load are FDA cleared or approved (Abbott Molecular, Des Plaines, IL), whereas the EBV viral load assay is a laboratory-developed test (analyte-specific reagents; Abbott Molecular). Samples are batched, and a negative, low-positive, and high-positive control are included with each run. Assay controls for CMV and HIV are in-kit controls provided by Abbott; assay controls for the EBV laboratory-developed test (LDT) were obtained from Exact Diagnostics (Ft. Worth, TX) and were validated by Michigan Medicine Clinical Microbiology Laboratory prior to assay implementation. Recalibration of assays was performed as appropriate in accordance with College of American Pathologists (CAP) checklist requirements (12).

### Management of assay control data.

Unity Real Time was implemented in the first quarter of 2019 in the Michigan Medicine Clinical Microbiology Laboratory as a tool to standardize the entering, tracking and management of QC data for CMV, EBV, and HIV viral load assays. This software allows user input of results for each control upon completion of testing for a run and determines acceptability based on user-defined criteria.

In routine clinical practice, control data were entered into Unity Real Time for each run and evaluated using 2-2S (2 consecutive values of the same control, or both controls simultaneously, >2 standard deviations [SDs] outside the mean) and 1-3S (1 control value >3 SDs outside the mean) QC rules based on an initial mean value of the controls and a fixed standard deviation based on validation data and historical performance. Clinical runs that triggered either of these rules were considered “failed,” and the entire run was repeated. Each viral load assay was assigned a unique identifier in Unity Real Time that specified the extraction (m2000 sp) and amplification (m2000 rt) instrument used, and only a single extraction-amplification instrument combination was used for each assay. Under each unique identifier, a separate QC data table was used for each lot of controls. Actions and comments were recorded by the bench technologist, including the dates of changes of reagent lots, recalibration of the instruments, and any instrument maintenance (repair or preventative) that was performed. Review of QC data was performed in accordance with the College of American Pathologists inspection checklist (12).

### Data analysis.

For this study, CMV, EBV, and HIV control values were retrospectively evaluated to compare viral load assays with various levels of regulatory approval, analytical refinement, and guidelines on quality assurance. To assess the performance of various viral load assays relative to the TEa (if known), data from the first lot of controls recorded in Unity Real Time was extracted. The data comprised the following: for HIV, 73 days of control values, from 15 December 2018 to 20 July 2019; for CMV, 77 days of control values, from 7 January 2019 to 4 April 2019; and for EBV, 83 days of control values, from 20 January 2019 to 25 April 2019. For the purposes of this analysis and to evaluate the intrinsic precision of the assays, initial means and fixed SDs were not applied; rather, Unity Real Time was used to retrospectively analyze the data to calculate means, SDs, CV, and 95% and 99% confidence intervals. However, since Unity Real Time calculates sigma values based only on accepted control values, a single 5+SD value from one rejected run was considered an outlier and removed from the HIV data set prior to analysis. Sigma metrics were calculated using the following formula: (TEa [%] − bias [%])/CV [%]. Bias was set to 0 in the calculation of sigma values, because we were interested in within-laboratory error and because single operational pathways (dedicated m2000sp and m2000rt instruments) were used for each viral load assay.

## RESULTS AND DISCUSSION

In this report, we show approaches taken to establish criteria for run acceptability, provide performance data, and demonstrate robustness of both *in vitro* diagnostic (IVD) and laboratory-developed viral load assays. Furthermore, we describe attributes of a commercially available software program (Unity Real Time) that simplifies and standardizes molecular QC data management while offering molecular laboratories a comprehensive tool for ensuring robust analytical performance.

**FIG 1:**
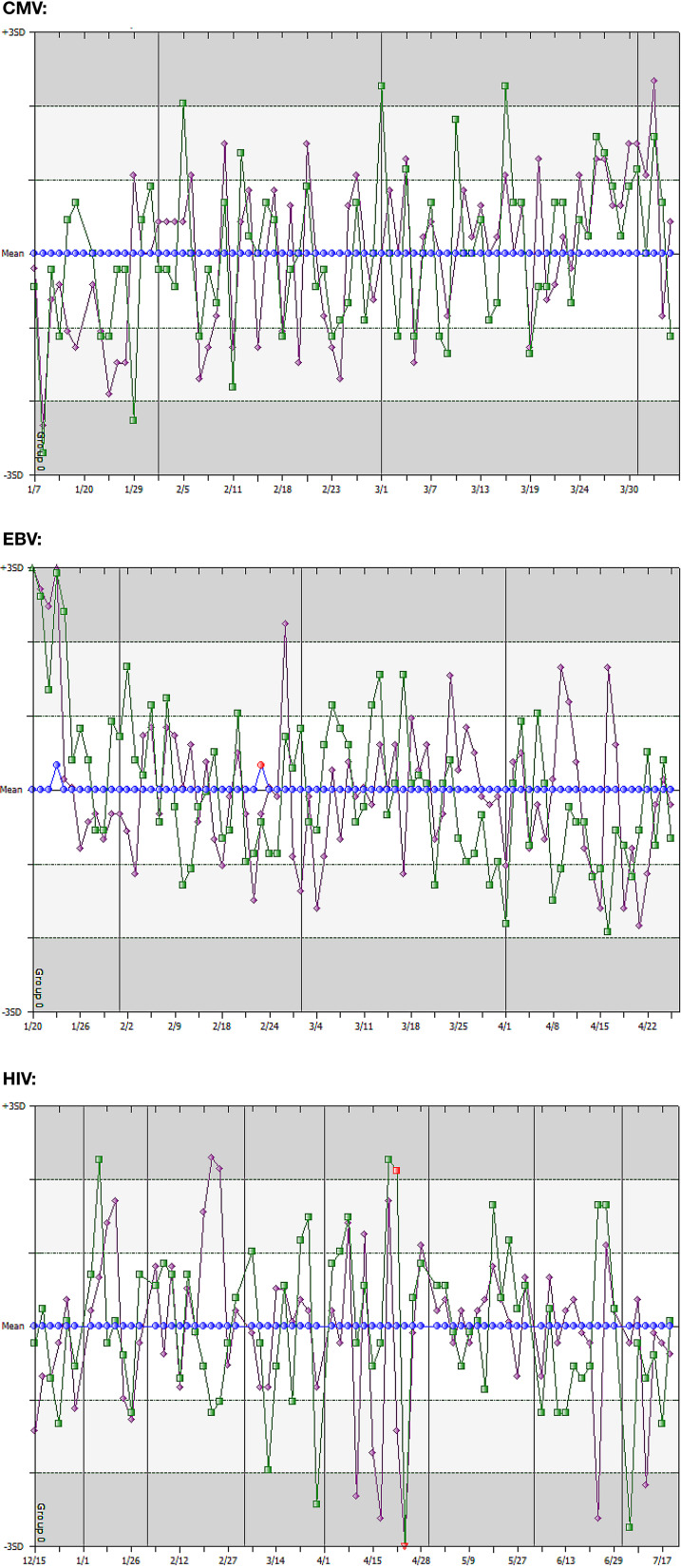
Trends of in-kit control values for CMV, EBV, and HIV viral load assays. Levey-Jennings charts of quality control data for level 1 (negative, blue circles), level 2 (low-positive, green squares), and level 3 (high-positive, purple diamonds) controls are centered on each control’s cumulative means ± SDs and plotted over time for CMV, EBV, and HIV. Horizontal lines indicate ranges for SDs. Individual control values for runs that were rejected based on laboratory criteria are indicated in red. The red value in the EBV graph was rejected due to a positive reaction in the negative control. The two red values in the HIV graph were rejected due to violations of the 1-3S rule.

To evaluate the precision and accuracy of each assay, level 1 (negative), level 2 (low-positive), and level 3 (high-positive) control values were entered in Unity Real Time for 77, 83, and 73 days of testing for CMV, EBV, and HIV viral load assays, respectively. The QC rules (1-3S and 2-2S) that were used in the clinical laboratory during the time frame of the data analysis were selected to be applied by Unity Real Time to determine acceptability of values obtained from each run. Figure 1 shows Levey-Jennings charts generated by Unity Real Time with the values centered at each control’s cumulative means from the total set of data, which allowed comparison of control performance over time. Controls that failed these rules, as applied to the data sets using means and ranges determined by Unity Real Time, identified runs that would have been rejected and repeated (Fig. 1, red symbols). While control values for HIV were relatively stable around the means, CMV level 2 and level 3 controls exhibited a downward trend early in the timeline and a slight upward trend late in the timeline. In contrast, the EBV assay exhibited a slight upward trend early and a slight downward trend late in the timeline of its data set. It is important to note that the time frames for the trends observed with the CMV and EBV controls did not overlap and were not of a magnitude that would have resulted in rejection of a run. These trends could have been the result of analytical changes in the reagents or control materials between lots or potential degradation over time. However, assay recalibration did not have an impact on these trends or rule violations in this data set (data not shown). While Unity Real Time is useful in tracking these trends, these scenarios provide some justification for the routine use of independent controls (complementing the in-kit or assay controls) with each run across lots to assist in troubleshooting sources of variation (6).

**FIG 2:**
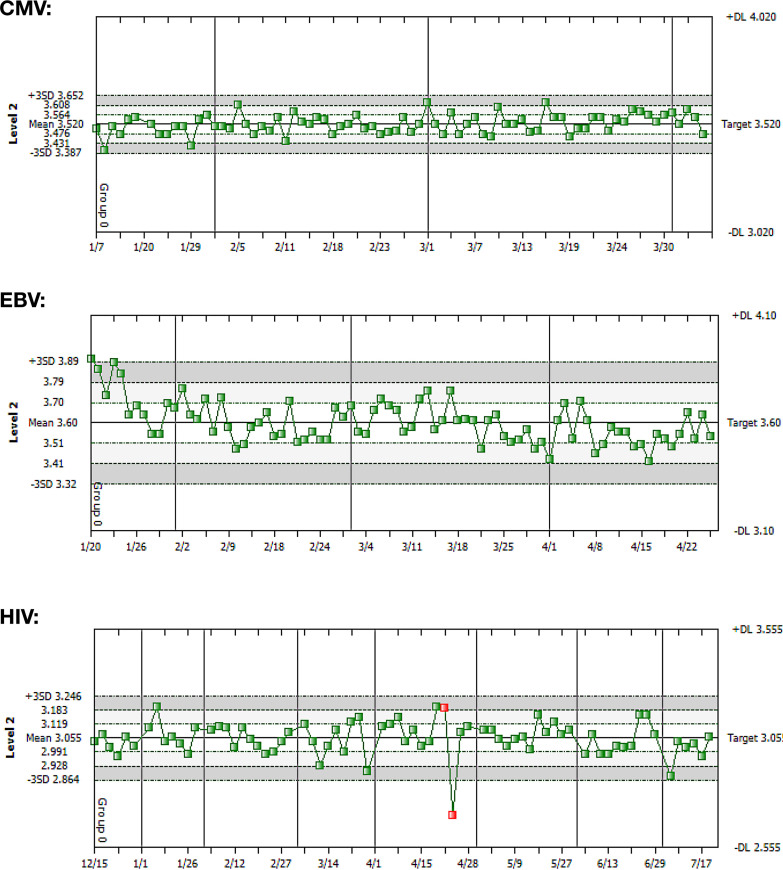
Performance of level 2 (low-positive) controls compared to TEa for CMV, EBV, and HIV viral load assays. Levey-Jennings charts have cumulative mean and standard deviations for CMV, EBV, and HIV level 2 controls on the left *y* axis of each panel. Horizontal lines indicate ranges for SDs. TEa for each control was set at 0.5 log_10_, and ranges are on the right *y* axis of each panel. Individual control values for runs that were rejected based on laboratory criteria are indicated in red. The first red value in the HIV graph was rejected due to violation of the 2-2S rule and the second one due to violation of the 1-3S rule.

Next, the performance of each assay was assessed in the context of clinical guidelines for monitoring of viral loads and treatment decisions. Based on these guidelines, a TEa of 0.5 log_10_ would ensure detection of clinically significant differences for HIV and CMV at viral load levels used to make decisions about therapy (7–11). Although the TEa required for effective monitoring of EBV viral load is less clear, the same value of 0.5 log_10_ was applied so that each assay could be compared directly. For these additional analyses, we chose to focus on the level 2 (low-positive) control, as the clinical decision points for each of the viral load assays included in this study were closer to those control values. Figure 2 shows Levey-Jennings charts centered on the cumulative means of the level 2 controls for each assay using TEa for the range. As indicated, each assay demonstrated a high level of precision with narrow SD ranges. Even results that failed 1-3S or 2-2S QC rules were well within the TEa and therefore clinically acceptable. Taken together, the results of these analyses indicate that the originally selected QC rules (2-2S and 1-3S) were overly sensitive and resulted in excess false rejections (i.e., the probability of false rejection [*P*_fr_] was too high). It is relevant to also note that the cumulative means of each control compared well to clinical decision points that are available through practice guidelines (7–11). For HIV, it is important to distinguish 2.3 log_10_ (200) copies/ml from <1.6 log_10_ (40) copies/ml, a 0.7 log_10_ difference, and the mean value for the HIV level 2 control was 3.06 log_10_ copies/ml. For CMV, guidelines are appropriately informed by the expected precision of the assay, such that a 0.5-log_10_ change is considered clinically significant, except below 3 log_10_ IU/ml, where a 0.7-log difference is considered significant. In our institution, 3.48 log_10_ IU/ml is the threshold used to start antiviral therapy and is near the mean of the level 2 control (3.52 log_10_ IU/ml).

To determine the performance of each assay relative to the TEa, Unity Real Time was used to calculate summary data from the level 2 QC data. For our purposes, we used 0.5 log_10_ copies/ml as the TEa for each assay and determined the CV from the cumulative data for each assay’s level 2 controls (Table 1). The 99% and 95% confidence intervals were also determined from the calculated means, SDs, and CVs. These data show that the log_10_ changes in viral load for each assay that could be detected with 95 and 99% confidence were well below the 0.5 log_10_ clinical requirement. This further indicated that the precision of each assay was sufficient to reliably detect a clinically significant difference in viral load.

**TABLE 1:** Sigma statistics for level 2 (low-positive) controls for each viral load assay

Assay	Log_10_ viral load^*a*^		CV (%)^*a*^	Sigma^*b*^	CI change	
	Calculated mean	SD			99%^*c*^	95%^*d*^
CMV	3.52	0.044	1.26	11.32	0.114	0.087
EBV	3.61	0.099	2.74	5.06	0.255	0.194
HIV	3.06	0.065	2.13	7.66	0.168	0.128

a Determined cumulatively from the entire data set; other calculations are based on this value.

b Unbiased sigma calculated with a TEa of 0.5 log_10_.

c Log_10_ viral load change that can be detected with 99% confidence.

d Log_10_ viral load change that can be detected with 95% confidence.

Although traditionally applied to non-health care settings, such as the manufacturing industry (13), application of Six Sigma principles is also becoming a best practice for health care and laboratory quality management improvement (3, 14). In a laboratory setting, calculation of the sigma metric allows quantification of the amount of variation in a process (in this case, a viral load assay) by determining the number of SDs between the test’s true value and the defined tolerance limit (3). Using Unity Real Time, sigma values were determined to be 11.32 for CMV, 7.66 for HIV, and 5.06 for EBV relative to a TEa of 0.5 log_10_ (Table 1).

Clinical laboratory standards dictate that processes that achieve >5 sigma are high-quality, high-performing tests (15), a value which each assay demonstrated. While the CMV (11.32 sigma) and HIV (7.66 sigma) assays performed well above the Six Sigma standard of “world class” performance, the EBV assay (5.06 sigma) still performed at a level that is considered “excellent” (3). Indeed, at a level of 5 sigma, one expects to see 230 defects (or false results) per million occurrences (or tests performed), or 1 in every 4,348 individual patient tests. Based on the Michigan Medicine laboratory test volume for EBV viral loads, this translates to an erroneous result in this assay approximately only once every 1.25 years if no QC rules were in place. At a level of 7.66 sigma, one expects 0.36 defect per billion occurrences (1 in every 2.78 billion individual patient tests); at a level of 11.32 sigma, one expects <0.01 defect per trillion occurrences (1 in every 10^14^ individual patient tests).

**FIG 3:**
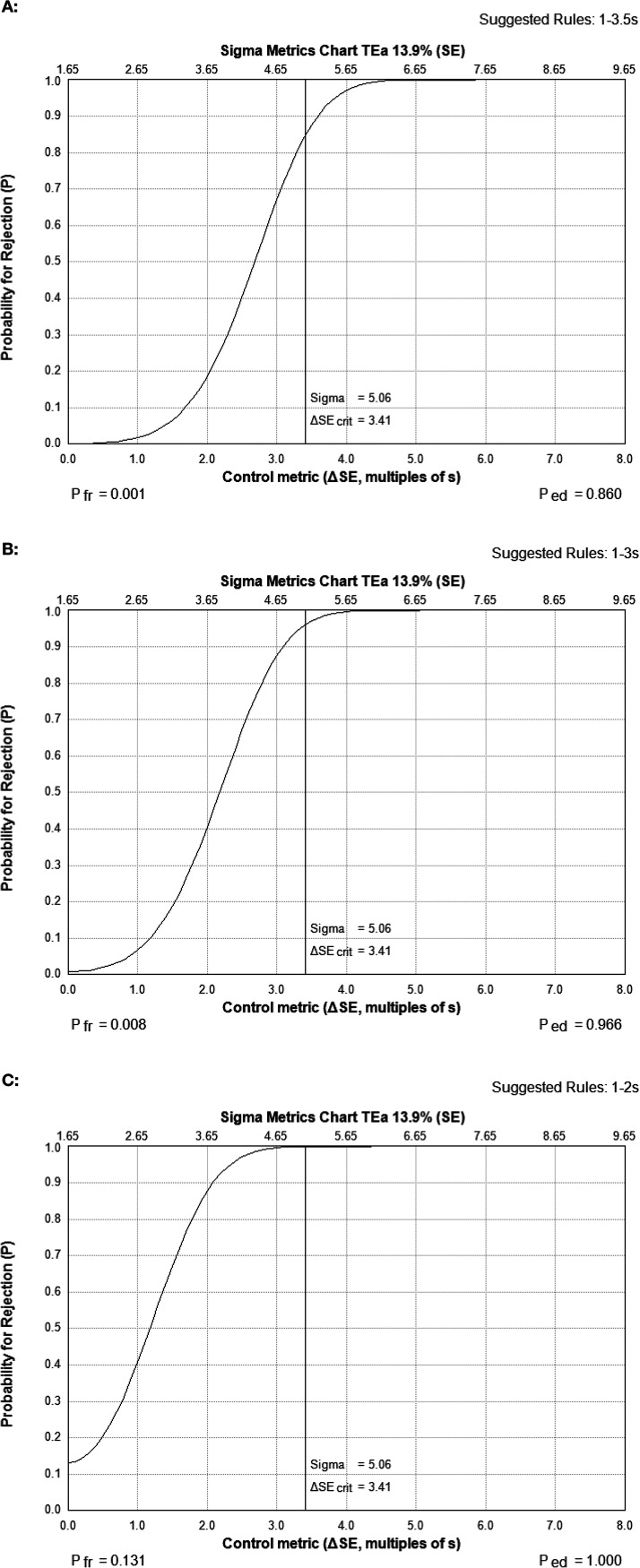
Impact of application of different Westgard rules on error detection and rejection rates for EBV viral load. The QC Design module in Unity Real Time calculated the probability of error detection and probability of false rejection for EBV viral load controls based on the sigma value of the level 2 (low-positive) control. Individual sigma metrics charts are shown for the 1-3.5S (A), 1-3S (B), and 1-2S (C) Westgard rules. *P*_ed_, probability of error detection; *P*_fr_, probability of false rejection; ΔSEcrit, critical increase in systematic error.

Finally, we used Unity Real Time to recommend QC rules based on the calculated sigma values. Prior to the implementation of Unity Real Time, Michigan Medicine Clinical Microbiology Laboratory routinely applied 1-3S (1 value >3 SDs from the mean) and 2-2S (2 consecutive values of the same control >2 SDs from the mean) QC rules to determine acceptability of assay runs. QC Design is a module in Unity Real Time that utilizes the QC data and derived sigma metrics to select the best QC rules to apply to an assay that maximize detection of errors while minimizing false rejection of small variations that are not expected to impact patient care. It is important to note, however, that these rules are valid only when applied to data with a normal distribution. Although each assay was shown to perform very well (with high sigma values), we focused on the EBV assay as the viral load assay with the lowest sigma value for its level 2 (low-positive) control to determine the effect of application of different QC rules on error detection and false rejection rates. Figure 3 shows the sigma metrics charts generated using the Unity Real Time QC Design module following application of the 1-3.5S (Fig. 3A), 1-3S (Fig. 3B), and 1-2S (Fig. 3C) QC rules. Application of the 1-3.5S rule provides a probability of error detection (*P*_ed_) of 0.860 (86.0%), with a very low probability of false rejection (*P*_fr_) of 0.001 (0.1%). Although application of the 1-3S rule provided a higher *P*_ed_ of 0.966, it also had a higher *P*_fr_ of 0.008 than 1-3.5S. Applying the 1-2S rule when running 3 levels of QC (negative, low-positive, and high-positive controls) in each run resulted in a 1.00 *P*_ed_, but the *P*_fr_ was 0.131 (13.1%) for the level 2 control, which would create a false rejection for more than 1 of each 10 QC results. As both the 1-3.5S and 1-3S rules have an acceptable analytical quality assurance (16), the 1-3.5S rule would be the preferred selection due to a lower *P*_fr_, although the difference is very small. Taken together, the data indicate that utilizing the rules suggested by Unity QC Design (based on the cumulative data) for EBV would result in an approximately 10-fold reduction in the rejection rates for these assays (*P*_fr_ reduction from 0.011 for the 1-3S/2-2S rule originally used in the laboratory to 0.001 for 1-3.5S) (data not shown). When applied to CMV and HIV (assays with sigma metrics of >7), Unity Real Time QC Design suggested 1-5S rules for both (data not shown). While the use of tighter QC rules can reduce the frequency of erroneous results, this should be balanced with having rules that are too stringent and increase the rate of false rejections (*P*_fr_). Similarly, achieving a high sigma metric demonstrates the safety of relaxing QC rules while still achieving appropriate rates of error detection (*P*_ed_). The implementation of QC rules that are informed by in-house precision data, TEa, and sigma values could increase efficiency and reduce costs (incurred by unnecessary repeats or reviews of results) by rejecting only runs with imprecision that might affect patient care.

There are limitations to the information presented which should be acknowledged: to evaluate the utility of Unity Real Time in monitoring data and recommending QC rules, we used data from a single lot of controls and calculated the SD intrinsic to that data set. Although standard clinical practice is to calculate means and SDs from 20 data points tested on different days (6), to assess the true SD of the system, we would also recommend assessing the SD across multiple lots of reagents. The SD from each lot could be used to generate recommended QC rules, and then a decision can be made based on which rule is consistently recommended. Historical SD data across multiple lots could also be used to assign a fixed SD for evaluation of daily control values. In addition, since monitoring of QC data over time serves as a proxy for overall system performance and error within individual patient samples cannot be measured, it is still possible to report erroneous patient results that are due to random error of a magnitude greater than the TEa. However, the probability of this is low in our assays due to the robustness of the test system with its established QC rules. Finally, this study assessed the performance of assays that utilize an external calibrator that is performed once per lot and applied to all specimens. This should detect changes in extraction efficiency, heat block performance for PCR cycling, or fluorescence detection. It is not known whether these results can be extrapolated to a different system where there is an internal calibrator in each sample and presents opportunities for further investigation.

### Conclusion.

A fundamental aspect of laboratory quality assurance (QA) programs is to ensure that the tolerance limits for their assays are appropriate so that the changes observed are clinically relevant and can be accurately and repeatedly detected. Ideally, there is a consensus on the total allowable error for an assay that supports clinical decision-making so that the QA program focuses on preventing significant errors. The laboratory can then assess the performance of the assay relative to the TEa and calculate a sigma metric, predicting how frequently an error will occur without QC. Finally, the laboratory can institute QC rules that balance error detection and false rejection. In this report, we show that, in our hands, assays to determine CMV, EBV, and HIV viral loads were able to perform within the 0.5 log_10_ TEa and operated at a high sigma value, suggesting that less restrictive QC rules could be implemented. The approach described in this study provides a framework for laboratories to establish similar assessments of assay and/or independent controls to routinely evaluate molecular viral load assay performance. Furthermore, we found that Unity Real Time is a robust, modular product that can support laboratory QC data management activities through its automated calculation tools and QC Design module and can be used in conjunction with the approaches described herein to standardize quality assurance activities for molecular assays.
